# Low quality antibody responses in critically ill patients hospitalized with pandemic influenza A(H1N1)pdm09 virus infection

**DOI:** 10.1038/s41598-022-18977-0

**Published:** 2022-09-02

**Authors:** Xiuhua Lu, Zhu Guo, Zhu-Nan Li, Crystal Holiday, Feng Liu, Stacie Jefferson, F. Liaini Gross, Wen-Ping Tzeng, Anand Kumar, Ian A. York, Timothy M. Uyeki, Terrence Tumpey, James Stevens, Min Z. Levine

**Affiliations:** 1 Influenza Division, National Center for Immunization and Respiratory Diseases, Centers for Disease Control and Prevention, MS H17-5, 1600 Clifton Road, Atlanta, GA 30329 USA; 2 Section of Infectious Diseases, University of Manitoba, Winnipeg, Canada

**Keywords:** Immunology, Diseases

## Abstract

Although some adults infected with influenza 2009 A(H1N1)pdm09 viruses mounted high hemagglutination inhibition (HAI) antibody response, they still suffered from severe disease, or even death. Here, we analyzed antibody profiles in patients (n = 31, 17–65 years) admitted to intensive care units (ICUs) with lung failure and invasive mechanical ventilation use due to infection with A(H1N1)pdm09 viruses during 2009–2011. We performed a comprehensive analysis of the quality and quantity of antibody responses using HAI, virus neutralization, biolayer interferometry, enzyme-linked-lectin and enzyme-linked immunosorbent assays. At time of the ICU admission, 45% (14/31) of the patients had HAI antibody titers ≥ 80 in the first serum (S1), most (13/14) exhibited narrowly-focused HAI and/or anti-HA-head binding antibodies targeting single epitopes in or around the receptor binding site. In contrast, 42% (13/31) of the patients with HAI titers ≤ 10 in S1 had non-neutralizing anti-HA-stem antibodies against A(H1N1)pdm09 viruses. Only 19% (6/31) of the patients showed HA-specific IgG1-dominant antibody responses. Three of 5 fatal patients possessed highly focused cross-type HAI antibodies targeting the (K130 + Q223)-epitopes with extremely low avidity. Our findings suggest that narrowly-focused low-quality antibody responses targeting specific HA-epitopes may have contributed to severe infection of the lower respiratory tract.

## Introduction

Infection with influenza virus causes substantial morbidity and mortality annually worldwide, despite the availability of the influenza vaccines and antiviral drugs^1^. Over the past century, influenza A viruses (IAV) have caused four pandemics, including 1918 A(H1N1), 1957 A(H2N2), 1968 A(H3N2), and 2009 A(H1N1)pdm09 pandemic^2^. The 2009 A(H1N1) pandemic resulted in an estimated 201,200 respiratory deaths globally; 80% of the deaths were in people younger than 65 years^3^. Currently, two IAV subtypes, A(H3N2), A(H1N1)pdm09, and two distinct lineages of influenza B viruses (IBV, Yamagata-lineage [B-Yam] and Victoria-lineage [B-Vic]) are circulating among humans.

Antibody responses to influenza virus infections are complex, involving neutralizing antibodies and non-neutralizing antibodies at systemic (serum) and respiratory levels^4–6^. Antibody responses also comprise different antibody isotypes and IgG subclasses targeting various viral epitopes, even for the same viral proteins with different antiviral mechanisms^4,5,7,8^. Not all antibodies contribute equally to protection; some antibodies have unknown or even adverse effects^6,9–13^. Dimeric secretory IgA antibodies provide most protection in the upper respiratory tract^14^. IgG1 is dominant in the lower respiratory tract, which is important for preventing influenza pneumonia^4,12^. In general, high-affinity neutralizing antibodies confer better protection than low-affinity neutralizing antibodies and non-neutralizing antibodies^9,10,12,15^. Neutralizing anti-stem antibodies and non-neutralizing antibodies have indirect antiviral effects via FcR-mediated effector functions and complement-mediated lysis for decreasing viral spread and attenuating disease^5–8,16,17^. Differences in these complex immune responses to influenza virus infection can have profound effects on disease severity and clinical outcome^18–20^.

Most protective antibodies induced by influenza virus infection target the major surface hemagglutinin (HA) glycoprotein^4,6,21^. HA is cleaved by proteases into HA1 and HA2 subdomains to yield infectious viruses^4,21^. The receptor-binding site (RBS) on immune-dominant globular head of HA1, including 130-loop, 150-loop, 190-helix and 220-loop, mediates binding to the host receptor. The HA2 plus the N and C termini of HA1 form the immune-subdominant stem domain mediates subsequent fusion^21–23^. The neutralizing antibodies targeting epitopes in or around the RBS, for blocking virus and sialic acid receptor binding, can be measured by both hemagglutination inhibition (HAI) assay and virus neutralization (VN) assay, while the neutralizing antibodies targeting the HA-stem domain, for preventing viral fusion and HA cleavage, can only be detected by VN assay^2,6,24^. The conserved RBS and stem region are targets for broadly neutralizing antibodies (bnAbs)^6,22–27^. Insertion, deletion, or mutations in HA-130 and/or HA-220 loop (H1 numbering) allow virus escape from the RBS-targeted bnAbs^21,25–28^.

Past exposures to A(H1N1) IAV can affect the subsequent response to A(H1N1)pdm09 virus in humans of different age groups. Some A(H1N1)pdm09 virus-infected patients born between 1983 and 1996 generated dominant HAI antibodies focusing on the K130-epitope^29^. Approximately 20–40% of A(H1N1)pdm09 vaccinated middle-aged adults born between 1961 and 1983 produced dominant HAI antibodies targeting the K163-epitope^30,31^, and were more susceptible to infection with recent A(H1N1)pdm09 viruses with the HA-K163Q mutation^32^.

An HAI antibody titer of 40 has historically been associated with a 50% reduction in the risk of influenza virus infection in adults^2^. However, in influenza vaccine trials conducted since 1943, a small number of patients from vaccine breakthrough cases had HAI antibody titers ≥ 40 (e.g. 40–2048) against IAV and IBV^33–36^. The reason for the failure of seemingly protective HAI antibody titers to provide protection has not been fully explored. Moreover, antibodies with higher HAI antibody titers but lower-avidity IgG to A(H1N1)pdm09 virus antigens were found in inpatients compared to outpatients with A(H1N1)pdm09 virus infections^19,37^.

Our previous study showed that some critically ill patients with A(H1N1)pdm09 virus infection had robust levels of HAI antibodies at admission to intensive care units (ICUs). Surprisingly, several patients with fatal outcomes had significantly higher HAI antibody titers than those who survived^38^. These unexpected results prompted our further investigation of the quality of antibody responses that correlate with protection from severe outcomes from A(H1N1)pdm09 virus infection. Here, we conducted a comprehensive analysis of antibody profiles in 31 ICU patients. We characterized the HAI, neutralizing, anti-HA-head/stem and anti-neuraminidase (NA) antibody responses using sera collected throughout the course of the illness. We also determined anti-HA antibody immunodominance and mapped epitopes of dominant HAI as well as anti-HA-head antibodies. Finally, we analyzed antibody isotypes and IgG subclass responses in these severely ill patients.

## Results

### Patient characteristics

Patients (n = 31, age range 17–65 years, median age 46 years), who were hospitalized in ICUs with laboratory confirmed A/California/07/2009-like (CA/09) A(H1N1)pdm09 virus infection in Canada between 2009 and 2011, were included in the current study. Patient characteristics are described in Table 1 and previous publications^38,39^. None of the patients received influenza vaccines. Most patients (81%) had common comorbidities, including chronic lung diseases (35%), obesity (65%), and/or pregnancy (10%). Five patients had fatal outcomes. Bacterial infections were identified in up to 29% of patients during 1–45 days post ICU admission (dpicu) and up to 40% of fatal cases during 10–25 dpicu. Sera were collected from 31 patients 1 time (n = 1), 2 times (n = 4), or ≥ 3 times (n = 26) during 1–30 dpicu and 2–45 days post-symptom onset (dpo).

**Table 1:** A(H1N1)pdm09 virus-infected critically ill patient characteristics.

Patient no. (#)^a^	Age (year)	Birth Year	sex	Laboratory confirmed infection^b^	Predisposing conditions^c^	Clinical characteristics	Days from symptom onset to serum collection	Days from ICU admission to serum collection	Days from symptom onset to ICU admission	Cilinical outcome (days from symptom onset to death)
#1*	**47**	**1962**	**Female**	**Serology**	**Obese (BMI = 58), asthma, others**^¥^	**Shock, b. pneumonia (25 dpicu)**^†^	**8, 9, 10, 12, 14, 21, 28**	**2, 3, 4, 6, 8, 15, 22**	**6**	**Died (34)**
#2*	**40**	**1969**	**Female**	**Virology**	**Obese (BMI = 31)**	**Shock**	**3, 4**	**3, 4**	**0**	**Died (17)**
#3	43	1966	Female	Virology	Obese (BMI = 35), asthma	None	18, 19, 20, 35, 41	1, 2, 3, 18, 24	17	Discharged
#4	27	1982	Female	Virology	Obese (BMI = 50)	Pulmonary edema fluid	13, 16, 28	7, 10, 22	6	Discharged
#5	53	1956	Female	Serology	Obese (BMI = 30)	Shock, b. pneumonia (23 dpicu)	18, 19, 31, 45	2, 3, 15, 29	16	Discharged
#6	46	1963	Male	Virology	Obese (BMI = 31)	b. pneumonia (45 dpicu)	8, 11, 14, 17	1, 4, 7, 10	7	Discharged
#7	50	1959	Male	Virology	Obese (BMI = 36)	Shock	3, 6, 8, 15, 22	2, 5, 7, 14, 21	1	Discharged
#8	46	1963	Male	Virology	None	None	9, 14	2, 7	7	Discharged
#9	23	1986	Female	Virology	None	b. pneumonia (8 dpicu)	13,14,15	2, 3, 4	11	Discharged
#10	53	1956	Male	Virology	Obese (BMI = 30), asthma	Shock	9, 10, 11, 14, 18	2, 3, 4, 7, 11	7	Discharged
#11*	**17**	**1992**	**Female**	**Virology**	**Pregnant (35 weeks)**	**Shock, ECMO use**	**9**	**2**	**7**	**Died (28)**
#12	53	1956	Female	Virology	Obese (BMI = 55), COPD	none	5, 7, 11, 14	1, 3, 7, 10	4	Discharged
#13	29	1980	Female	Virology	Immunosuppression, chemotherapy	Shock, b. pneumonia (1 dpicu)	25, 30, 34, 40	5, 10, 14, 20	20	Discharged
#14	35	1974	Male	Serology	None	None	2, 3, 4, 5, 7, 10	2, 3, 4, 5, 7, 10	0	Discharged
#15	57	1952	Male	Virology	None	None	7, 9, 13, 27	2, 4, 8, 22	5	Discharged
#16	51	1958	Female	Virology	Obese (BMI = 46)	None	6, 8, 12	1, 3, 7	5	Discharged
#17	24	1985	Female	Virology	Obese (BMI = 40)	Shock, b. pneumonia (2 dpicu)	7, 8, 9, 11, 19	3, 4, 5, 7, 15	4	Discharged
#18	40	1969	Female	Virology	Obese (BMI = 46), chemotherapy	Shock, b. pneumonia (1 dpicu)	6, 8, 9, 11, 34	2, 4, 5, 7, 30	4	Discharged
#19*	**42**	**1967**	**Male**	**Virology**	**Obese (BMI = 33), renal condition**	**Shock, b. pneumonia (10 dpicu)**	**7, 13**	**3, 9**	**4**	**Died (16)**
#20	52	1957	Male	Virology	Obese (BMI = 38), asthma	None	3, 4, 5, 7, 9	1, 2, 3, 5, 7	2	Discharged
#21	56	1953	Female	Virology	Obese (BMI = 37), COPD	None	7, 8, 10, 12	4, 5, 7, 9	3	Discharged
#22	55	1954	Female	Virology	None	None	17, 18, 20, 29, 36	3, 4, 6, 15, 22	14	Discharged
#23	31	1978	Male	Virology	Obese (BMI = 41), asthma	Pulmonary edema fluid	10, 13, 15, 22, 29	2, 5, 7, 14, 21	8	Discharged
#24	55	1954	Female	Virology	Obese (BMI = 42), asthma	None	8, 9, 11, 13, 27	1, 2, 4, 6, 20	7	Discharged
#25*	**65**	**1944**	**Female**	**Serology**	**Obese (BMI = 30)**	**Shock**	**8, 9, 11, 13, 16**	**2, 3, 5, 7, 10**	**6**	**Died (16)**
#26	58	1953	Male	Virology	None	Shock, b. pneumonia (1 dpicu)	8, 9, 10, 12, 14, 21	2, 3, 4, 6, 8, 15	6	Discharged
#27	41	1968	Female	Virology	Obese (BMI = 42), asthma	Pulmonary edema fluid	5, 7, 9, 12, 16, 23	4, 6, 8, 11, 15, 22	1	Discharged
#28	36	1973	Female	Virology	Asthma	None	4, 5, 6, 8, 10	2, 3, 4, 6, 8	2	Discharged
#29	39	1970	Female	Virology	Pregnant, asthma	None	5, 6, 8, 10, 16	2, 3, 5, 7, 13	3	Discharged
#30	22	1987	Female	Virology	Pregnant	None	7, 9	2, 4	5	Discharged
#31	52	1957	Female	Virology	Obese (BMI = 57)	None	4, 5, 6, 8, 10, 17, 24	1, 2, 3, 5, 7, 14, 21	3	Discharged

^¥^Others: patient #1 (chronic lung/renal/cardiovascular condition, diabetes mellitus, hematologic malignancy).

*Fatal cases in bold. ^†^Bacterial pneumonia (b. pneumonia) was detected 1–45 days post ICU admission (dpicu).

^a^Patients were admitted to ICU between 2009 (n = 29) and 2011 (n = 2, #21 and #26). All patients, except #20, received invasive mechanical ventilation use. The patients did not receive influenza vaccine.

^b^Infection with wt-CA/09-like viruses were confirmed by virology (n = 27, RT-PCR and/or virus isolation) or serology (n = 4, ≥ fourfold rise in HAI titers to wt-CA/09).

^c^Obese, Body mass index (BMI) ≥ 30; COPD, chronic obstructive pulmonary disease; ECMO, extra-corporal membrane oxygenation.

### Most patients had low quality anti-HA antibody responses at ICU admission

**Figure 1:**
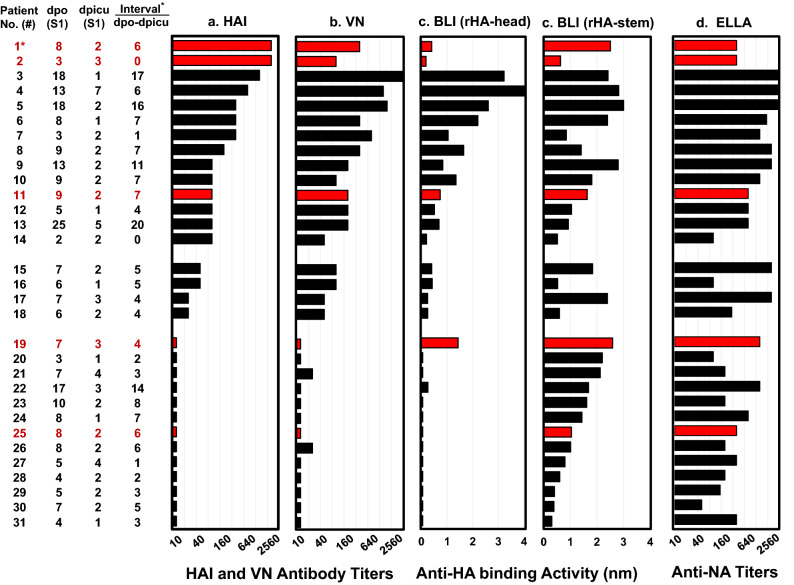
A(H1N1)pdm09 virus infections induced low quality antibody responses in most patients at the time of ICU admission. The first serum samples (S1) collected 1 to 7 days post ICU admission (dpicu) and 2 to 25 days post-symptom onset (dpo) from 31 patients were tested by: (**a**) HAI assays using wt-CA/09 (Q223), (**b**) VN assays using MX/09 (Q223QR); (**c**) BLI assays using rHA-head from wt-CA/09 and rHA-stem from A/Michigan/45/2015, and (**d**) ELLA assays using A(H6N1) reassortant virus possessing wt-CA/09 NA. Antibody responses are illustrated by black bars for survivors and red bars for patients with fatal outcomes. For each sample, we completed three independent HAI and VN assays. ELLA assays were performed in duplicate. BLI assays were performed in 2 independent assays. *The interval between S1 dpo and S1 dpicu, and fatal patients in red.

To investigate whether the quality of antibody responses was associated with severe clinical outcomes, we analyzed serum antibody profiles using HAI, VN, biolayer interferometry (BLI) and enzyme-linked lectin assay (ELLA). We aligned HAI, VN, anti-HA-head, anti-HA-stem, and anti-NA antibody results in the first serum collections (S1) (Fig. 1). Forty-five percent of patients (14/31, #1–#14) had HAI antibody titers in S1 ≥ 80 against CA/09 at 1–7 dpicu (2–25 dpo). Strikingly, two deceased patients (#1 and #2) had extremely high HAI antibody titers (S1 = 2560) but very low anti-rHA-head antibody binding activities (ABA) of 0.4 nm at 2 dpicu (8 dpo) and 0.2 nm at 3 dpicu (3 dpo), respectively as determined by BLI assays. In contrast, one deceased patient #19 exhibited high anti-rHA-head ABA (S1 = 1.4 nm) and anti-rHA-stem ABA (S1 = 2.6 nm) but had HAI and VN antibody titers ≤ 10 at 3 dpicu (7 dpo). Another deceased patient #25 displayed only non-neutralizing anti-rHA-stem ABA (S1 = 1 nm) at 2 dpicu (8 dpo). Furthermore, positive correlations between VN antibody titers and anti-rHA-stem ABAs were not observed in the 17 patients (#15–#31) possessing HAI antibody titers in S1 ≤ 40, such as #15 and #16 (VN = 80, anti-stem ABAs = 0.5–1.8 nm), #17 and #18 (VN = 40, anti-stem ABAs = 0.6–2.4 nm), and #19 to #31 (VN ≤ 20, anti-stem ABAs = 0.3–2.6 nm). This suggests that most (94%) of these patients (#15, #17–#31) had dominant antibody responses that targeted HA-stem non-neutralizing epitope(s) at 1–4 dpicu (3–17 dpo). Unexpectedly, most patients (21/31, 68%), including 5 fatal patients, also displayed high neuraminidase inhibition (NAI) antibody titers (S1 ≥ 320) against CA/09 NA (Fig. 1d).

These data suggested that infections with CA/09-like viruses induced low quality anti-HA antibody responses during the early clinical course in most (61%) of the ICU patients.

### Kinetics of antibody responses against A(H1N1)pdm09 virus antigens

While in the ICU, antibody increases from S1 to later serum samples were observed in many patients (HAI ≥ 80: from 45 to 84%, VN ≥ 160: from 39 to 81%, NAI ≥ 320: from 68 to 94%, and anti-rHA ABA ≥ 1 nm: from 55 to 94%) (Table 2 and Supplementary Fig. 1). Five patients [#19 (fatal), #20, #21, #28, and #30] showed low or no HAI and VN antibody titers (HAI: ≤ 10–40 and VN:20–80) at 4–9 dpicu (9–13 dpo), although 3 of them (#21, #28, and #30) showed substantial increases in anti-rHA-head ABAs and anti-rHA-stem ABAs.

**Table 2:** A(H1N1)pdm09 virus-infected critically ill patient antibody responses.

Patient	Samples		HAI^a^	VN^b^	ELLA^c^		BLI^d^	
no. (#)	dpo^†^	dpicu^†^	CA/09	MX/09	H6N1	rHA	rHA-head	rHA-stem
#1*	**8**	**2**	**2560**	**320**	**320**	**1.8**	**0.4**	**2.5**
	**28**	**22**	**2560**	**5120**	**5120**^**‡**^	**4.2**	**4.6**	**3.9**
#2*	**3**	**3**	**2560**	**80**	**NT**	**0.7**	**0.2**	**0.6**
	**4**	**4**	**2560**	**80**	**320**	**0.3**	**0.3**	**0.6**
#3	18	1	1280	6400	5120	2.9	3.2	2.4
	41	24	1280	6400	5120	3.6	3.8	2.8
#4	13	7	640	1280	5120	3.9	4.0	2.8
	28	22	1280	2560	5120	3.9	4.4	2.7
#5	18	2	320	1600	5120	3.1	2.6	3.0
	45	29	320	2560	5120	2.9	2.5	2.7
#6	8	1	320	320	1920	2.4	2.2	2.4
	14	7	2560	5120	5120	3.4	3.5	2.6
#7	3	2	320	640	1280	1.2	1.0	0.8
	22	21	12,800	25,600	5120	3.6	3.8	2.7
#8	9	2	160	320	2560	1.6	1.6	1.4
	14	7	5120	12,800	5120	3.6	5.2	2.6
#9	13	2	80	160	2560	2.2	0.8	2.8
	15	4	320	640	5120	2.8	1.9	3.3
#10	9	2	80	80	1280	1.6	1.3	1.8
	18	11	1280	2560	5120	3.8	3.9	3.1
#11*	**9**	**2**	**80**	**160**	**640**	**1.2**	**0.7**	**1.6**
#12	5	1	80	160	640	0.8	0.5	1.0
	14	10	640	640	5120	2.7	2.9	2.2
#13	25	5	80	160	640	1.1	0.7	0.9
	40	20	640	1280	1920	2.8	2.3	2.3
#14	2	2	80	160	80	0.5	0.3	0.5
	3	3	1280	320	80	0.9	0.6	0.8
#15	7	2	40	80	2560	1.4	0.5	1.8
	27	22	640	3200	5120	3.1	2.3	2.7
#16	6	1	40	80	80	0.6	0.4	0.5
	12	7	640	5120	5120	3.5	4.1	2.4
#17	7	3	20	40	2560	1.8	0.2	2.4
	11	7	320	1280	5120	3.3	2.7	3.0
#18	6	2	20	40	240	0.6	0.2	0.6
	34	30	2560	5120	5120	3.4	3.7	2.5
#19*	**7**	**3**	**< **	**< **	**1280**	**2.1**	**1.4**	**2.6**
	**13**	**9**	**40**	**80**	**3840**	**1.5**	**0.6**	**3.8**
#20	3	1	<	<	80	1.5	<	2.2
	9	7	<	20	160	1.4	<	2.1
#21	7	4	<	20	160	1.4	<	2.1
	12	9	20	40	640	2.6	1.6	2.5
#22	17	3	<	<	1280	1.0	0.3	1.7
	36	22	640	1600	5120	3.2	3.0	2.7
#23	10	2	<	<	160	0.8	<	1.6
	29	21	1280	3200	5120	3.7	4.1	2.8
#24	8	1	<	<	640	0.8	<	1.4
	27	20	2560	6400	5120	4.2	3.8	3.4
#25*	**8**	**2**	**< **	**<**	**320**	**0.3**	**<**	**1.0**
	**13**	**7**	**5120**	**1280**	**5120**	**2.7**	**2.2**	**2.7**
#26	8	2	<	20	160	0.5	<	1.0
	21	15	640	1280	5120	3.7	2.4	3.0
#27	5	4	<	<	320	0.5	<	0.8
	23	22	320	1280	3840	3.0	2.8	2.2
#28	4	2	<	<	160	0.4	<	0.6
	10	8	20	40	2560	2.9	2.1	2.7
#29	5	2	<	<	120	0.2	<	0.4
	16	13	1280	2560	5120	3.8	4.4	2.7
#30	7	2	<	<	40	0.3	0.1	0.4
	9	4	<	20	320	1.3	0.6	1.8
#31	4	1	<	<	320	0.1	<	0.3
	24	21	320	1280	5120	3.5	3.1	2.8

We completed 3 independent HAI and VN assays. BLI and ELLA assays were performed in duplicate.

*Fatal patients in bold; <, titers below 20 in HAI assays and VN assays or ABA < 0.1 nm in BLI assays.

^†^dpo, days post-symptom onset; ^†^dpicu, days post ICU admission; ^‡^Anti-NA titers ≥ 5120; NT, not tested.

^a^HAI antibody titers detected by HAI assays using wt-CA/09 (Q223) virus.

^b^Neutralizing antibody (VN) titers detected by VN assays with MX/09 (Q223QR mixture) virus.

^c^Anti-NA antibody titers detected by ELLA assays using H6N1 virus possessing wt-CA/09 NA.

^d^Anti-HA ABA detected by BLI assays using rHA and rHA-head from wt-CA/09, and rHA-stem from A/Michigan/45/2015, respectively.

**Figure 2:**
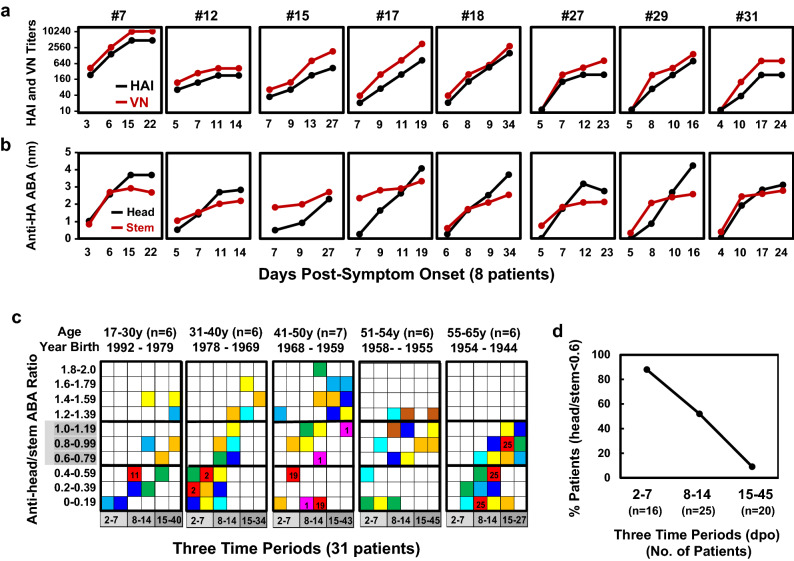
Kinetics of anti-HA antibody responses. Serum samples were tested by HAI assays using wt-CA/09, VN assays using MX/09, and BLI assays using rHA-head from wt-CA/09 and rHA-stem from A/Michigan/45/2015. (**a**) HAI and VN antibody response kinetics in 8 patients are illustrated by black lines and red lines, respectively. (**b**) Anti-HA-head and anti-HA-stem antibody response kinetics are illustrated by black lines and red lines, respectively. (**c**) Ratio of anti-head/stem ABA in 31 patients was categorized into 5 age-groups. Each colored square in 5 age-groups represents the ratio in each serum sample. Each color(yellow, orange, blue, dark blue, green, cyan, brown, red, and pink) in each age group represented the ratio(s) from the same patient’s serum sample(s) that were collected 1–4 times at 3 different time periods of 2–7 dpo, 8–14 dpo, and 15–45 dpo. Red and pink square with patient numbers represented 5 fatal patients; other color squares without patient numbers represented 26 surviving patients. (**d**) Summary of the percentage of 31 patients with ratio of anti-head/stem ABA < 0.6. We completed 3 independent HAI and VN assays. BLI assays were performed in 2 independent assays.

Kinetics of anti-HA antibody responses against A(H1N1)pdm09 virus antigens in 8 patients with multiple days of serum collections available (S1 at ≤ 7 dpo and the last samples at ≥ 14 dpo) were analyzed (Fig. 2a,b). HAI and VN antibody titers in S1 were similar (within twofold) and increased at a similar rate. In general, VN antibody titers were two- to four-fold higher than HAI antibody titers in later serum samples, consistent with antibody responses in mildly ill patients infected with CA/09-like viruses during the 2009 A(H1N1) pandemic^40^. Seven of 8 patients (except #7) exhibited higher anti-rHA-stem ABAs than anti-rHA-head ABAs in S1 (4–7 dpo), these patients had much sharper increase of anti-rHA-head ABAs than anti-rHA-stem ABAs from S1. After 1–3 weeks, 7 of 8 patients (except #15), displayed higher anti-rHA-head ABAs than anti-rHA-stem ABAs, and all 8 patients survived.

Next, we compared antibody immunodominance to HA-head versus HA-stem in 31 patients by calculating the ratio of anti-rHA-head/anti-rHA-stem ABAs (Fig. 2c,d). We considered the arbitrary ratios < 0.6 and ≥ 1.2 as indications that the patient possessed dominant anti-HA-stem or anti-HA-head antibodies, respectively. Fourteen of 16 patients (88%), who provided S1 at 2–7 dpo, displayed ratios < 0.6, in contrast, only 2 patients (#7 and #16) showed ratios of 1.2 and 0.8 at 2–7 dpo, respectively. Interestingly, all 5 patients suffered fatal outcomes displayed ratios < 0.6 in S1 (3–9 dpo). The ratio of anti-rHA-head/anti-rHA-stem ABAs increased over time in most patients. Only 2 of 20 (10%) patients (#9 and #22), who provided sera at 15–45 dpo, showed ratios < 0.6, whereas 10 of 20 (50%) patients (#3, #4, #6, #7, #10, #17, #18, #23, #27, #29) exhibited ratios ≥ 1.2 at 15–45 dpo. These data indicated that shifting of antibody immunodominance from HA-stem to HA-head occurred in most patients while in ICU (Fig. 2).

### Infections with CA/09-like viruses induced focused HAI and anti-HA-head binding antibody responses in most critically ill patients

To determine whether patients developed focused HAI antibody responses targeting specific epitopes from infections, 4 reverse genetics (RG) viruses were generated, including viruses possessing wild-type (wt) CA/09 HA (wt-CA/09), wt-CA/09 with HA-K163Q mutation (RG-K163Q), wt-CA/09 with double mutations at D127N + N129T (addition of a glycosylation motif, RG-127gly), and wt-CA/09 with HA-K130 deletion (RG-130del). We performed HAI assays using 4 RG-viruses and 2 egg-grown viruses: X-179A (A/California/07/2009-PR8 with Q223R and K209T egg-adapted mutations) and MX/09 (A/Mexico/4108/2009 with Q223QR mixture) (Supplementary Table 1). Focused HAI antibody was defined by ≥ fourfold reduction in HAI antibody titers to virus-mutant(s) compared to wt-CA/09. We found that 19 of 26 (73%) patients, who provided sera with HAI antibody titers of ≥ 80, had focused HAI antibody responses targeting 6 different epitopes possessing K130 + Q223, 127gly + K130, 127gly + Q223, 127gly, K130, or K163 (Table 3 and Supplementary Fig. 2). Interestingly, the deceased patient #1 showed a shifting of dominant antibody targeting (K130 + Q223)-epitope at 8 dpo to K163-epitope at 14–28 dpo. Importantly, nearly all HAI antibodies that only focused on single epitopes were detected in S1 of 3 deceased patients (#1, #2, and #11). Deceased patient #25 showed only non-neutralizing anti-stem ABA at 2 dpicu (8 dpo) (Fig. 1), then developed focused HAI antibody with extremely high HAI antibody titers (5120) at 7 dpicu (13 dpo). It is worth noting that 3 patients with fatal outcomes (#1 [8 dpo], #2 [3 and 4 dpo], #25 [13 dpo]) had highly focused HAI antibodies targeting (K130 + Q223)-epitopes (Table 3).

**Table 3:** CA/09-like virus-infection induced focused HAI antibody responses.

Patient	Sera		HAI^a^							Fold reduction^b^			Focused HAI antibody responses
			CA/09	MX/09	X-179A	RG	RG	RG	X-179A	RG	RG	RG	
No. (#)	dpo^†^	dpicu^†^	wt	Q223QR	Q223R	K163Q	127gly	130del	Q223R	K163Q	127gly	130del	
#1*	**8**	**2**	**2560**	**40**	**< **	**1280**	**2560**	**80**	**256**	**–**^**‡**^	**–**	**32**	**K130, Q223**
	**14**	**8**	**160**	**40**	**40**	**< **	**160**	**160**	**4**	**16**	**–**	**–**	**K163, Q223**
	**28**	**22**	**2560**	**2560**	**640**	**160**	**2560**	**2560**	**4**	**16**	**–**	**–**	**K163, Q223**
#2*	**3**	**3**	**2560**	**160**	**80**	**2560**	**2560**	**160**	**32**	**–**	**–**	**16**	**K130, Q223**
	**4**	**4**	**2560**	**160**	**80**	**2560**	**2560**	**160**	**32**	**–**	**–**	**16**	**K130, Q223**
#3	18	1	1280	1280	1280	1280	1280	160	–	–	–	8	K130
	35	18	1280	1280	1280	1280	1280	160	–	–	–	8	K130
	41	24	1280	1280	1280	1280	1280	160	–	–	–	8	K130
#4	13	7	640	320	640	320	40	40	–	–	16	16	127gly, K130
	16	10	1280	1280	1280	1280	160	160	–	–	8	8	127gly, K130
	28	22	1280	1280	1280	1280	160	160	–	–	8	8	127gly, K130
#5	18	2	320	320	320	320	80	160	–	–	4	–	127gly
	45	29	640	640	640	640	80	160	–	–	8	4	127gly, K130
#6	8	1	320	320	80	320	320	80	4	–	–	4	K130, Q223
	11	4	1280	640	320	1280	1280	320	4	–	–	4	K130, Q223
	14	7	2560	2560	640	1280	1280	640	4	–	–	4	K130, Q223
#7	3	2	320	160	160	320	160	320	–	–	–	–	ND^¥^
	22	21	12,800	12,800	12,800	12,800	12,800	12,800	–	–	–	–	ND
#8	9	2	160	160	80	40	80	160	–	4	–	–	K163
	14	7	5120	2560	5120	1280	5120	5120	–	4	–	–	K163
#9	13	2	80	80	40	80	80	40	–	–	–	–	ND
	15	4	320	320	320	320	320	160	–	–	–	–	ND
#10	9	2	80	80	80	80	<	<	–	–	8	8	127gly, K130
	14	7	640	1280	1280	640	80	80	–	–	8	8	127gly, K130
	18	11	1280	1280	1280	1280	160	160	–	–	8	8	127gly, K130
#11*	**9**	**2**	**80**	**80**	**80**	**80**	**80**	**< **	**–**	**–**	**–**	**16**	**K130**
#12	5	1	80	40	40	80	20	80	–	–	4	–	127gly
	7	3	160	160	80	320	40	320	–	–	4	–	127gly
	14	10	640	320	160	640	80	640	4	–	8	–	127gly, Q223
#13	25	5	80	80	80	20	80	80	–	4	–	–	K163
	30	10	320	320	320	40	320	320	–	8	–	–	K163
	40	20	640	640	640	80	640	640	–	8	–	–	K163
#14	2	2	80	40	20	80	80	20	4	–	–	4	K130, Q223
	4	4	1280	160	20	1280	1280	40	64	–	–	32	K130, Q223
	10	10	1280	80	40	1280	1280	40	32	–	–	32	K130, Q223
#15	13	8	320	320	320	320	80	160	–	–	4	–	127gly
	27	22	640	640	640	640	80	160	–	–	8	4	127gly, K130
#16	8	3	320	160	160	160	160	160	–	–	–	–	ND
	12	7	640	640	640	640	640	640	–	–	–	–	ND
#17	9	5	80	80	80	80	40	20	–	–	–	4	K130
	11	7	320	320	320	320	160	40	–	–	–	8	K130
	19	15	320	320	320	320	160	40	–	–	–	8	K130
#18	8	4	160	160	160	160	160	160	–	–	–	–	ND
	34	30	2560	1280	1280	1280	1280	1280	–	–	–	–	ND
#22	20	6	320	320	160	160	160	320	–	–	–	–	ND
	36	22	640	640	640	640	320	640	–	–	–	–	ND
#23	15	7	640	640	320	640	80	640	–	–	8	–	127gly
	22	14	1280	1280	1280	1280	160	1280	–	–	8	–	127gly
	29	21	1280	1280	1280	1280	320	1280	–	–	4	–	127gly
#24	11	4	160	80	160	160	40	20	–	–	4	8	127gly, K130
	13	6	640	1280	640	640	160	40	–	–	4	16	127gly, K130
	27	20	2560	2560	2560	2560	640	160	–	–	4	16	127gly, K130
#25*	**13**	**7**	**5120**	**640**	**320**	**5120**	**5120**	**640**	**16**	**–**	**–**	**8**	**K130, Q223**
#26	12	6	80	160	160	160	160	80	–	–	–	–	ND
	21	15	640	640	640	640	640	320	–	–	–	–	ND
#27	7	6	160	160	160	20	160	160	–	8	–	–	K163
	23	22	320	160	320	40	320	320	–	8	–	–	K163
#29	10	7	320	320	160	80	160	160	–	4	–	–	K163
	16	13	1280	1280	640	320	640	640	–	4	–	–	K163
#31	17	14	320	320	320	320	160	160	–	–	–	–	ND
	24	21	320	320	320	320	160	160	–	–	–	–	ND

^†^dpo, days post-symptom onset; ^†^dpicu, days post ICU admission; ^‡^–, ≤ twofold reductions; ^¥^ND, not detected.

*Fatal patients in bold. For each sample, we completed three independent HAI assays.

^a^Sera from 26 patients possessing HAI titers of ≥ 80 against wt-CA/09 were tested by HAI assays with the 6 viruses.

^b^Fold reduction of HAI titers against virus-mutant(s) compared to wt-CA/09.

**Figure 3:**
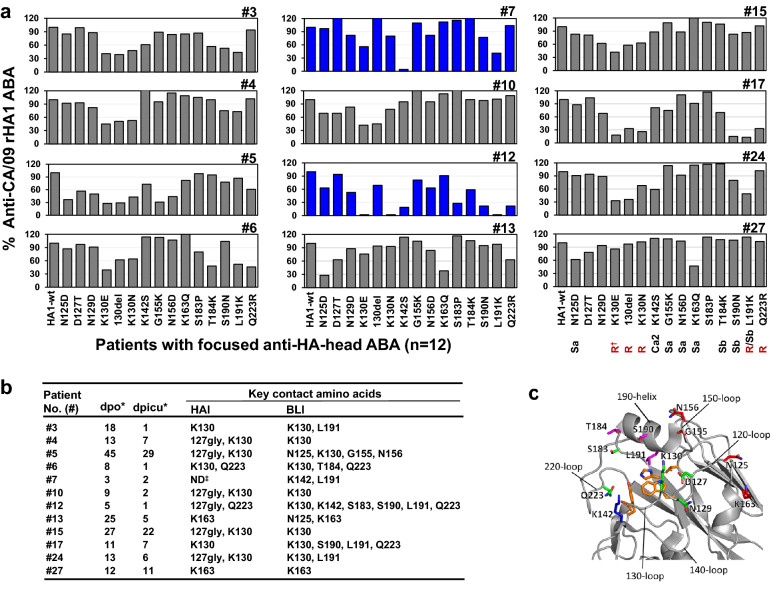
Determination of immunodominance of anti-HA-head binding antibody responses and epitope mapping. Anti-HA-head ABAs were determined by BLI assays using a rHA1-wt (HA-head from CA/09) and 15 rHA1-mutants possessing single point mutations or a K130 deletion (130del). (**a**) The 12 patients displayed focused anti-head ABA [defined by > 50% ABA reduction against rHA1-mutant(s) compared to rHA1-wt]. Patients #7 and #12 showing completely focused ABA are highlighted in blue. (**b**) Summary of key contact aa determined by HAI assays and BLI assays. Key contact aa are determined as virus-mutants or rHA1-mutants causing ≥ fourfold HAI antibody reduction in HAI assays or > 50% ABA reduction in BLI assays, respectively. (**c**) Key contact residues are mapped onto the CA/09 HA-head structure monomer. Antigenic sites Sa (red), Sb (Magenta), Ca (Blue), Receptor binding site (RBS, orange). *Serum collection days post-symptom onset (dpo) and post ICU admission (dpicu). ^†^R, RBS. ^‡^ND, not determined.

To map the epitopes of focused anti-HA-head binding antibodies, we created a rHA1-wt (HA1 from CA/09) and 15 rHA1-mutants for BLI assays (Supplementary Table 1). Here, focused anti-HA-head binding antibodies were defined by > 50% reduction of ABA to anti-rHA1-mutant(s) compared to rHA1-wt. Of 22 patients tested, 55% (n = 12) were found to possess focused anti-HA-head ABAs (Fig. 3), and the remainder 10 patients did not show such focused antibody responses (Supplementary Table 2). Sera from 9 patients, including 5 who died, were not tested in BLI assay, either due to low ABAs to rHA1-wt or insufficient volume of serum (Table 2 and Supplementary Table 2). Notably, patients #7 and #12 displayed completely focused anti-rHA-head ABAs targeting (K142 + L191)-epitope at 3 dpo and (K130 + K142 + S183 + S190 + L191 + Q223)-epitope at 5 dpo, respectively (Fig. 3). The same dominant antibodies were found in later serum collections for these two patients (15–22 dpo [#7] and 7–14 dpo [#12], Supplementary Table 2).

Taken together, we found that most patients who had HAI antibody titers of ≥ 80 at various time points post ICU admission presented or developed focused HAI and/or anti-HA-head binding antibodies targeting different epitopes including the 120-loop, 130-loop 140-loop, 150-loop, 160-loop, 190-helix, and/or 220-loop. Almost all patients who suffered fatal outcomes (except #19) had extremely focused HAI antibody responses.

### Some patients displayed dominant anti-CA/09 HAI antibodies cross-reactive with A(H3N2) IAV or IBVs

Next, we investigated specificity of HAI antibodies in 26 patients, who had HAI antibody titers of ≥ 80 to wt-CA/09 (Supplementary Table 3). Sera were tested by HAI assays against wt-CA/09, 7 epidemiologically important A(H1N1) viruses circulated between 1977 and 2007 [A/USSR/90/1977 (USSR/77), A/England/333/1980 (ENG/80), A/Taiwan/1/1986 (TW/86), A/Texas/36/1991 (TX/91), A/New Caledonia/20/1999 (NC/99), A/Solomon Islands/3/2006 (SI/06), and A/Brisbane/59/2007 (BR/07)], one A(H3N2) IAV [A/Brisbane/10/2007 (BR/10)] and one IBV [B/Brisbane/60/2008 (BR/60)]. BR/10 and BR/60 circulated at very low levels during the 2009 A(H1N1)pdm09 pandemic. Most patients (18/26, 69%) showed ≥ fourfold antibody increases for wt-CA/09 and one or more 1977–2007 A(H1N1) viruses during their stay in ICU. Four patients also showed ≥ eightfold HAI antibody increase for BR/10 A(H3N2) IAV(#6 and #29) or BR/60 IBV (#14 and #25). Additionally, two deceased patients (#1 [8 dpo] and #2) showed high HAI antibody titers for both wt-CA/09 and BR/60 IBV (Supplementary Table 3). All these 6 patients, except #29, displayed dominant HAI antibodies targeting (K130 + Q223)-epitopes (Table 3). Among them, patients #1, #2, and #25 had fatal outcomes (Table 1).

**Figure 4:**
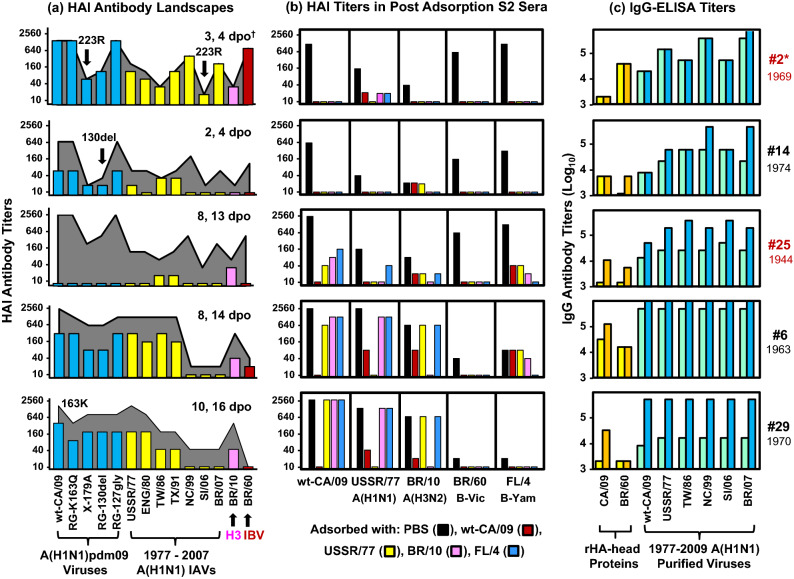
HAI antibodies targeting epitopes possessing HA-(K130 + Q223) cross-reacted with A(H3N2) IAV or IBVs. (**a**) HAI antibody landscapes in 5 patients were constructed using HAI assays with the 14 indicated viruses. HAI titers in S1 were shown in different colored bars: blue bars for wt-CA/09 and CA/09-mutants, yellow bars for 1977–2007 A(H1N1), pink bars for BR/10 A(H3N2), and red bars for BR/60 IBV. HAI antibody titers in the second sera are shown in gray landscapes. (**b**) Sera were adsorbed with purified viruses or PBS as a control. Post adsorption sera were tested by HAI assays with wt-CA/09, USSR/77, BR/10, BR/60 and FL/4. Antibody titers are expressed as color bars: post-adsorbed with PBS in black, wt-CA/09 in red, USSR/77 in yellow, BR/10 in pink, and FL/4 in blue. We completed two independent antibody adsorption assays. (**c**) Paired sera were tested by IgG-ELISA using two rHA-heads from CA/09 or BR/60 and 6 purified A(H1N1) viruses. IgG titers are shown in yellow or light blue bars for S1 and orange or dark blue bars for the second sera. We completed two independent ELISA assays. *Patient number (fatal patients in red) with birth year. ^†^Serum collection days post-symptom onset (dpo).

Next, we constructed HAI antibody landscapes for 5 patients, who displayed high or significant HAI antibody increases for BR/10 A(H3N2) IAV or BR/60 IBV, against 14 viruses (Fig. 4a). Patients #2 (3–4 dpo), #14 (4 dpo), and #25 (13 dpo) showed similar antibody landscapes; interestingly, these patients showed ≥ eightfold reduced HAI antibody titers to not only X-179A (Q223R) and RG-130del but also SI/06 (possessing Q223R mutation) compared to wt-CA/09 (Q223). Similar antibody landscape was also observed in S1 of patient #1 (Table 3 and Supplementary Table 3). In contrast, patients #6 and #29 showed different antibody landscapes, that displayed significant antibody increases for wt-CA/09, BR/10 A(H3N2) IAV but not for BR/60 IBV.

These ICU patients did not receive influenza vaccines before the illness. To determine whether simultaneous increases of HAI antibody titers to BR/10 or BR/60 were caused by co-infections with these viruses, we performed antibody adsorption assays using purified viruses of wt-CA/09, USSR/77, BR/10, B/Florida/04/2006 IBV (FL/4, B-Yam) and PBS as a control (Fig. 4b). HAI antibodies against wt-CA/09, BR/60 and FL/4 in deceased patients #2 and #14 were completely adsorbed not only by wt-CA/09, USSR/77 and FL/4 but also by BR/10 virus, suggesting that patients #2 and #14 possessed highly focused cross-type antibodies. HAI antibody against BR/60 in deceased patient #25 (13 dpo) was also completely adsorbed by all the viruses used; however, antibodies against wt-CA/09 were only partially adsorbed by USSR/77, BR/10, or FL/4, suggesting that patient #25 possessed mixed populations of HAI antibodies. Serum from patient #1 (S1, 8 dpo) was not tested due to insufficient volume. Patient #6 possessed mixed populations of HAI antibodies: dominant-antibody targeting (K130 + Q223)-epitope that cross-reacted between wt-CA/09 and BR/10 A(H3N2), and another antibody population cross-reactive for wt-CA/09 and USSR/77 (Fig. 4a,b). Patient #29 also possessed at least 2 populations of HAI antibodies: dominant-antibody targeting K163-epitope that cross-reacted for wt-CA/09 and USSR/77, and another antibody population that cross-reacted with wt-CA/09 and BR/10 (Fig. 4a,b).

Lastly, we tested the sera in IgG-ELISA assays using CA/09 rHA-head, BR/60 rHA-head, and 6 purified viruses (Fig. 4c). Patients #2, #14, and #25 displayed ≥ fourfold higher IgG titers to USSR/77, NC/99, and BR/07 A(H1N1) viruses compared to wt-CA/09 virus. Deceased patient #2 also had eightfold higher IgG antibody titers for BR/60 rHA-head compared to CA/09 rHA-head. In contrast, patients #6 and #29 had lower IgG antibody titers for BR/60 rHA-head compared to CA/09 rHA-head and equal IgG antibody titers for wt-CA/09 and1977-2007 A(H1N1) viruses (Fig. 4c).

Taken together, our data indicated that infection with CA/09-like viruses induced cross-subtype, even cross-type HAI antibody responses targeting (K130 + Q223)-epitope(s) in some ICU patients. However, only cross-type antibodies displayed lower IgG antibody titers for wt-CA/09 compared to pre-pandemic A(H1N1) viruses and/or rHA-head of IBV. Such low-avidity cross-type antibodies likely contributed to fatal outcomes in some patients.

### Most ICU patients did not exhibit IgG1-dominant serum antibody responses

**Figure 5:**
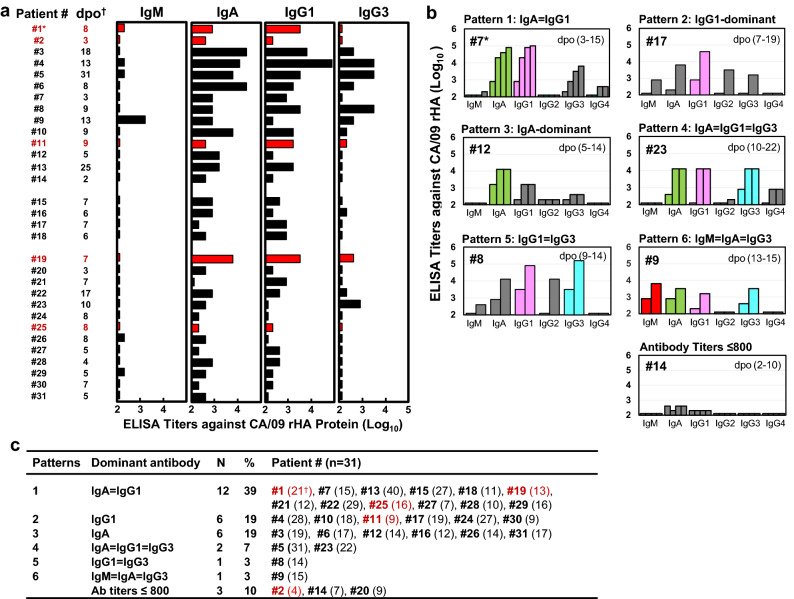
CA/09 HA-specific antibody isotype and IgG subclass responses. Sera were tested by ELISA using rHA from wt-CA/09. (**a**) IgM, IgA, IgG1, and IgG3 antibody responses in 31 patients. ELISA antibody titers are illustrated by black bars for survivors and red bars for fatal patients. (**b**) Representative patients showed 6 different antibody isotype and IgG subclass immunodominance response patterns. Antibody titers are shown in gray bars, but dominant and co-dominant isotype and IgG subclass are highlighted in other colors: IgG1 in pink, IgG3 in cyan, IgA in green, and IgM in red. (**c**) Summary of antibody isotype and IgG subclass response patterns at indicated serum collection time in 31 patients (Supplementary Table 4). *Fatal patients in red, ^†^serum collection days post-symptom onset (dpo). For each serum sample, we completed two independent ELISA assays.

Antibody isotypes and IgG subclasses were tested with sera from 31 patients by ELISA using CA/09 rHA (Fig. 5 and Supplementary Table 4). We found that 13 patients showed very low IgG1 titers (S1 ≤ 200); 9 patients had ≥ fourfold higher IgA than IgG1; patient #9 possessed mainly IgM and IgA in their S1 (Fig. 5a). Nevertheless, significantly increased (≥ fourfold) IgM (n = 8, 26%), IgA (n = 20, 65%), IgG1 (n = 23, 74%), IgG2 (n = 8, 26%), IgG3 (n = 18, 58%), and IgG4 (n = 5, 16%) antibody responses were observed while patients were in the ICU (Supplementary Table 4). Next, we analyzed immunodominance of antibody isotypes and IgG subclasses. We considered antibody isotype/IgG subclass antibody titers showing ≥ fourfold higher than others as dominant-antibodies. Examples of 6 immunodominance patterns were presented in Fig. 5b. Three patients (#2, #14, and #20) displayed low ELISA titers (≤ 800), and patients (n = 28) were grouped into 6 different patterns based on the sera collected at the indicated time (Fig. 5c and Supplementary Table 4). Only 6 (19%) patients exhibited IgG1-dominant responses. Most patients (68%) were IgA-dominant (n = 6), or IgA co-dominant with other isotype/IgG subclasses (n = 15). Four patients showed different immunodominance patterns in S1 and later serum samples, however, most patients displayed consistent patterns during the illness (Supplementary Table 4). Antibody isotypes and IgG subclasses analyses further indicated that antibody quality varied among patients.

## Discussion

Our findings provide insight into serum antibody profiles of patients with severe influenza A(H1N1)pdm09 virus infection during 2009–2011. At ICU admission, low-quality antibody responses, including extremely focused HAI antibody responses targeting specific epitopes on HA-head, non-neutralizing antibody responses targeting HA-stem, and/or low titers of HA-specific IgG1, were detected in these patients (Figs. 1, 2, 3, 4, 5 and Tables 2, 3). In addition, high titers but very low-avidity RBS-targeted antibodies that cross-reacted with influenza B viruses were detected in most patients with fatal outcome (Tables 1, 2, 3 and Fig. 4). Consistent with previous studies^18,39^, bacterial pneumonia was identified in only 9 patients, including 4 patients at 1–2 dpicu and 5 patients at 8–45 dpicu (Table 1). Therefore, low quality antibody responses particularly at early stage of illness may have contributed to the severe lung infections and fatal outcomes in most of these patients, although secondary bacterial infections also increased morbidity and mortality in some patients.

CA/09-like virus infection induced focused HAI and anti-HA-head binding antibody responses targeting in or around the RBS in 65% of patients (Table 3, Fig. 3, and Supplementary Fig. 2). Notably, 4 patients (#2, #7, #12, and #14) exhibited nearly all focused HAI antibodies or completely focused anti-rHA-head binding antibodies as early as 1–3 dpicu (2–5 dpo). Although A(H1N1)pdm09 virus shedding was detected in some patients with high HAI antibody titers (data not shown), these viruses were unfortunately not sequenced. Thus, whether such focused neutralizing antibodies could select escape mutants to evade host immunity is unknown. However, selection of escape mutants by human monoclonal antibodies (hmAbs) targeting the epitopes involving D127, K130, G155, K163, and by human anti-sera possessing focused K163-antibodies has been reported^9,41–44^. Highly focused antibody responses targeting epitopes that are absent on infecting viruses or newly formed escape mutants may not be able to aid in the protection against further infection, virus clearance from infected cells, and attenuation of disease severity^31,32^.

Fulminant influenza with acute respiratory failure as early as 0 dpo were observed in deceased patient #2 with mild obesity and patient #14 without any comorbidity (Table 1). Two patients did not display concurrent or secondary bacterial pneumonia (Table 1). Focused low-avidity HAI antibodies targeting the epitopes possessing HA-(K130 + Q223) were detected in 2 patients as early as 2–3 dpo (Fig. 1, Tables 2, 3). Surprisingly, the (K130 + Q223)-antibodies cross-reacted with both B-Yam and B-Vic IBVs (Fig. 4). The low-avidity (K130 + Q223)-antibodies were also detected in other 2 deceased patients (#1 and #25) (Tables 1, 2, 3, and Supplementary Table 3). To our knowledge, such RBS-targeting cross-type cross-reactive antibodies that could contribute to detrimental clinical outcomes have not been previously reported^45^, although a few rare human memory B cell clones which produced cross-type IgG have been isolated^27^. Nonetheless, some cross-subtype RBS-targeted hmAbs have been characterized; these hmAbs penetrated the RBS pocket using their unusually long HCDR3 loop to block virus attachment by direct competition with sialic acid host receptors^25–27^. If the binding affinity between the antibody and the viral RBS was lower than the binding affinity between the viral RBS and its natural sialic acid receptors on host cells, failure of antibody-mediated protection may occur. Notably, patients #2 and #14 had HAI antibody titers of ≥ 1280 as early as 3 dpo (Table 2), suggesting that these (K130 + Q223)-antibodies did not prevent infection from CA/09-like viruses. Our data also indicated that HAI and VN assays can effectively detect very low-avidity antibodies likely with no protective function in humans (Table 1 and Fig. 1). Thus, other immunological measures should also be considered to assess the antibody quality and correlates of protection.

Seven critically ill patients displayed focused HAI antibodies targeting the epitopes shielded by glycosylation at HA-127 (Table 3 and Supplementary Fig. 2). The hmAb EM4C04, which was isolated from a previously healthy adult with severe A(H1N1)pdm09 virus infection, can select escape mutants possessing HA-D127E change; such mutant viruses displayed altered receptor specificity and enhanced virulence in mice^24,42^. Any emergence of HA-127 mutation in A(H1N1)pdm09 viruses and their effect on viral pathogenicity should be closely monitored in humans.

Some patients displayed various levels of non-HAI and non-neutralizing antibodies only targeting HA-stem in S1 samples (Fig. 1). One deceased patient #19 exhibited high levels of non-neutralizing antibodies in S1 (7 dpo) targeting both HA-head and HA-stem domains (Fig. 1). Although neutralizing anti-HA-head antibodies can provide protection against influenza in humans, and neutralizing anti-HA-stem antibodies can provide protection against influenza in mice and ferrets in an Fc-receptor mediated manner^6,7,16,22,23^, it has also been reported that some non-neutralizing anti-HA-head and anti-HA-stem antibodies can enhance viral fusion activity and lead to antibody-dependent enhancement (ADE) of pneumonia disease in mice and pigs^11,13^. Therefore, high-affinity neutralizing antibodies often are beneficial, but some non-neutralizing antibodies may exacerbate the disease.

Influenza virus infection and vaccination usually induce dominant-IgG1 responses, which is important for preventing influenza pneumonia^4,46–48^. Surprisingly, only 19% of the patients exhibited dominant-IgG1 responses (Fig. 5). Most patients showed dominant-IgA or IgA co-dominant with IgG1, IgG3 and/or IgM (Fig. 5). Serum IgA cannot be transported into the respiratory secretions^14,49,50^. The role of serum IgA in protection and pathogenesis of lung disease is still poorly understood. Additionally, some patients displayed high levels of IgG3 (Fig. 5 and Supplementary Table 4), that have been associated with ADE disease in other viral infections^51^.

Anti-NA antibodies can reduce disease severity^2,36^. Unexpectedly, most the patients had high levels of NAI antibodies, even in those with fatal outcomes (Fig. 1 and Table 2). Our previous study indicated that some cross-reactive anti-NA antibodies induced by historical A(H1N1) viruses failed to reduce disease severity against novel IAV in mice^52^. More studies are needed to understand why high NAI antibodies failed to prevent severe disease in these patients.

At ICU admission, comorbidities were present in 81% of the patients, including obese, chronic lung disease, immunosuppression, and/or pregnancy (Table 1). Comorbidities such as obesity can have negative impact on virus-induced innate and adaptive immunity^53–55^. However, the 20 obese patients showed similar levels of anti-HA antibody responses as compared to the 11 non-obese patients (Table 1 and Supplementary Fig. 1). Overall, the levels of HAI and VN antibody responses in the most ICU patients with comorbidities were higher than those in the non-ICU patients infected with 2009 A(H1N1)pdm09 virus^40^, and most non-ICU patients did not have comorbidities. These data suggested that antibody quality but not quantity play an important role for disease severity.

There are several limitations in this study. First, it is challenging to collect multiple time-points of sera from ICU patients, therefore we were only able to include a small number of ICU patients in our current study, which did not allow further statistical analysis. Second, serum samples from age-matched mildly ill patients collected during 2009 A(H1N1)pdm09 pandemic were not available for comparison. Third, immune cells and other clinical samples were not collected in 2009. Thus, whether the lack of activation of other antiviral responses, such as reduced levels of IFNs, aberrant cell-mediated immunity, or exaggerated expression of proinflammatory cytokines and chemokines which can play critical roles in disease severity^18,20^, were not investigated here. Finally, the role of ADE should be considered in future antibody-quality study.

In summary, we observed multiple forms of low quality anti-HA antibody responses in severely ill patients infected with A(H1N1)pdm09 virus, especially during early stage of illness onset. Some patients showed extremely immunodominant HAI antibodies with very low-avidity or targeting the specific epitopes that are likely associated with selecting escape mutants. Others displayed dominant non-neutralizing antibodies with possibility of ADE of lung disease. Some patients also displayed IgA-dominant, but not IgG1-dominant antibody responses. Therefore, we conclude that low quality and/or narrowly focused antibody responses to CA/09 HA, especially during the early stage of the clinical course, along with comorbidities have contributed to severe infection of lung and progression to severe influenza. More studies are needed to advance our understanding of overall antibody quality in patients with different severity to inform the development of improved antibody-based immunotherapies and universal vaccines against influenza.

## Material and methods

### Patient enrollment and serum collection

During 2009 and 2011, Canadian ICU physicians established a multicenter cohort of critically-ill adolescents and adults hospitalized with laboratory-confirmed influenza A(H1N1)pdm09 virus infection^39^. Blood samples were collected when patients stayed at the ICUs (Table 1). All thirty-one patients (17–65 years old) admitted to ICUs with RT-PCR or serology confirmed influenza A(H1N1)pdm09 virus infection with available stored sera (at − 20 °C) were included in the current study. All patients provided informed consent for specimen collection and storage of sera for future analysis, informed consent was obtained from legal guardian(s) of the deceased. The study was approved by the National Center for Immunization and Respiratory Diseases, Centers for Disease Control and Prevention human subject research determination ethic committee review. All methods were carried out in accordance with relevant guidelines and regulations^56,57^.

### Influenza viruses

All viruses were propagated in embryonated eggs. Some viruses were purified on a liner sucrose gradient. Four viruses were generated by reverse genetics (RG), including the virus possessing wt-CA/09 HA, NA and 6 A/Puerto Rico/8/1934 (PR8) internal genes^30^. All viruses were sequenced, details of RG-viruses information are described in Supplementary Table 1.

### Hemagglutination inhibition (HAI) assay

Sera were treated with receptor-destroying enzyme (RDE, Denke-Seiken, Japan) to remove non-specific inhibitors, and adsorbed with packed turkey red blood cells (TRBCs) to remove non-specific agglutinins prior to testing with 4 HA units of virus and 0.5% TRBCs (World Health Organization manual).

### Traditional virus neutralization (VN) assay

Two-fold dilutions of RDE-treated sera were incubated with 100 TCID_50_ of virus at 37° for 1 h. Madin-Darby Canine Kidney (MDCK) cell monolayers in 96-well plates were washed 3 times with phosphate buffered saline (PBS). After 1 h, the virus-serum mixtures were supplemented with 1 µg/ml Tosyl phenylalanyl chloromethyl ketone (TPCK)-treated trypsin and 1% bovine serum albumin (BSA) were added to MDCK cells and incubated at 37 °C for 3 days. The neutralization antibody titer is the highest serum dilution demonstrating complete neutralization in which no HA titer in supernatants was detected.

### Antibody adsorption

Serum was mixed with ~ 10^5^ HAU of purified virus or PBS as a control. After incubating for about 2 h at 4 °C, the virus-serum mixture was centrifuged for 45 min at 100,000*g* to remove virus-antibody complexes and most of the unbound viruses. Residual viruses were removed by the addition of 100 µl of packed TRBCs^30^.

### Enzyme-linked immunosorbent assay (ELISA)

The 96-well plates were coated with 1 µg/ml rHA, 0.6 µg/ml rHA1 or 1000 HAU/ml of purified virus and were incubated at 4 °C overnight. Plates were blocked with 0.05% Tween-20 and 4% BSA in PBS for 1 h. Two-fold serially diluted RDE-treated sera were added to the plates and incubated for 2 h. The plates were washed three times with 0.05% Tween-20 in PBS. Horseradish peroxidase (HRP)-conjugated anti-human IgG, IgG1, IgG2, IgG3, IgG4, IgM, or IgA were added. Plates were incubated for 1 h, and then washed five times before adding OPD. Optical density (OD) measurements were taken at 490 nm. The ELISA antibody titer is the highest serum dilution where OD > three-fold background OD.

### Enzyme-linked lectin assay (ELLA)

NA inhibition (NAI) antibodies were detected using ELLA as described previously^58^. H6N1 reassortant virus with N1 from CA/09 and a mismatched HA from A/turkey/Massachusetts/3740/1975 H6 virus was used. Briefly, sera were first heat inactivated. Serial twofold diluted sera were then incubated with A(H6N1) virus in plates coated with fetuin for 16–18 h. Following incubation, HRP-labeled peanut agglutinin (lectin) was added to the reaction and incubated for 2 h, followed by tetramethylbenzidine (TMB) substrate to reveal enzymatic cleavage of fetuin by viral NA. The percent inhibition of NA enzymatic activity was calculated by comparing with values from virus control wells. Endpoint NAI antibody titers were calculated as the reciprocal of the highest dilution with at least 50% inhibition.

### Biolayer interferometry (BLI) assay

Full length HA ectodomain (residues 18–518, rHA from CA/09) were expressed and purified as described previously^59^. HA1 domain (residues 18–311, rHA-head from CA/09) was synthesized and sub-cloned into pIEx-4 vector. All subsequent HA1 mutants for epitope mapping were generated from the pIEx-4-HA1 clone (see Supplementary materials). HA-stem domain (residues 1–33, 312–386, and 420–501 from A/Michigan/45/2015 A(H1N1)pdm09 virus) with the linkers for the GEN4 construct was expressed and purified as described^60^. Determination of anti-rHA, anti-rHA-head, anti-rHA-stem antibody binding activity (ABA) was performed on an Octet Red instrument (Pall ForteBio, CA) according to the manufacturer’s instructions (see Supplementary materials). The use of BLI methodology to evaluate antibody avidity analysis has been described previously^59^.

## Supplementary Information

Supplementary Information.

## Data Availability

Data supporting the finding of the study are available from the corresponding author upon reasonable request.
