# Supplementary material for: Low quality antibody responses in critically ill patients hospitalized with pandemic influenza A(H1N1)pdm09 virus infection
Source: Sci Rep. 2022 Sep 2;12:14971. doi: 10.1038/s41598-022-18977-0 (PMC9440095; doi:10.1038/s41598-022-18977-0)
Supplement: Supplementary file 1 — Supplementary Information. [file 41598_2022_18977_MOESM1_ESM.pdf]

Supplementary Materials

Low Quality Antibody Responses in Critically Ill Patients Hospitalized with Pandemic Influenza A(H1N1)pdm09 Virus Infection

Xiuhua Lu,[a†] Zhu Guo,[a†] Zhu-Nan Li,[a] Crystal Holiday,[a] Feng Liu,[a] Stacie Jefferson,[a] F Liaini Gross,[a] Wen-Ping Tzeng,[a] Anand Kumar,[b] Ian A. York,[a] Timothy M. Uyeki,[a] Terrence Tumpey,[a] James Stevens,[a] and Min Z. Levine[a#]

Influenza Division, National Center for Immunization and Respiratory Diseases, Centers for Disease Control and Prevention, USA[a] and Section of Infectious Diseases, University of Manitoba, Winnipeg, Canada[b]

† These authors contributed equally to this work. Author order was determined on the basis of contributions to manuscript preparation.

# Corresponding author:

Min Z. Levine, Ph. D, MS H17-5, 1600 Clifton Road, Atlanta, Georgia 30329, USA

Tel: (404)-639-3504. E-mail: mlevine@cdc.gov

The running title: Low Quality Antibodies in Critically Ill Influenza Patients

Key words: Influenza, Critical illness, Human serum antibody responses, Immunodominance, Epitope mapping, Antibody binding activity, Antibody isotypes/IgG subclasses.

Supplementary Methods

Recombinant HA (rHA), rHA-head (HA1), and rHA-stem cloning and expression.

Full length HA ectodomain (residues 18-518, rHA) of CA/09 was expressed and purified as described previously (1). A codon optimized cDNA encoding the HA1 domain (residues 18-311) of CA/09 was synthesized (GenScript USA Inc., NJ) and sub-cloned into pIEx-4 vector (EMD Millipore, MA) using the In-Fusion HD cloning system (Clontech, CA). All subsequent HA1 mutants for epitope mapping were generated from this wild type pIEx-4-HA clone using the QuickChange Lightning Site-Directed Mutagenesis Kit (Stratagene, CA). The resulting constructs were transiently transfected into Spodoptera frugiperda Sf9 cells (EMD Millipore, MA) using the Cellfectin II transfection reagent (Life Technologies, NY). All procedures were performed following protocols provided by manufacturers. The transfected cells were grown in suspension on an orbital shaker at 27℃ for five days. All rHA1 proteins contained a signal sequence for secretion, a thrombin site at the C-terminus followed by a trimerizing sequence (foldon) from the bacteriophage T4 fibritin for generating functional trimers, and a histag to aid detection. The expression levels of rHA1s secreted in the culture supernatant were quantitated by chemiluminescent western blot analysis using anti-His antibody (Qiagen, CA) and a ChemiDoc MP imaging system (Bio-Rad, CA) following manufacturer directions. Concentrations of mutant rHA1 determined by densitometric analysis were normalized to the wild type and similar amount of rHA1s were applied in epitope mapping analysis without further purification. A codon optimized cDNA encoding the HA-stem domain (residues 1-33, 312-386, 420-501) of the mature HA gene of A(H1N1)pdm09 virus A/Michigan/45/2015 with the linkers for the GEN4

construct, as described by Yassine et al (2) was synthesized (GenScript USA Inc., NJ). The gene was sub-cloned into a pAc-GP67 baculovirus transfer vector. The final construct contained a signal sequence for secretion, a thrombin site at the C-terminus followed by a foldon sequence for generating functional trimers, and a histag. The rHA-stem was expressed in Hi5 cells and purified by standard histag purification and size exclusion chromatography.

Biolayer interferometry (BLI) assay.

Anti-rHA, anti-rHA-head, anti-rHA-stem antibody binding activity (ABA), and epitope mapping of dominant anti-head antibodies were performed by BLI assays on an Octet Red instrument (Pall ForteBio, CA) according to the manufacturer’s instructions. Briefly, rHA proteins was coupled to anti-penta-His biosensors by incubating the tip of biosensors into the supernatant of rHA proteins in kinetics buffer with the usage of a sidekick biosensor immobilization station (Pall ForteBio, CA). Sera were diluted in kinetics buffer, and binding was analyzed by BLI on an Octed Red instrument. Data were analyzed using the system software and fitted to a 1:1 binding model.

Supplementary Figures

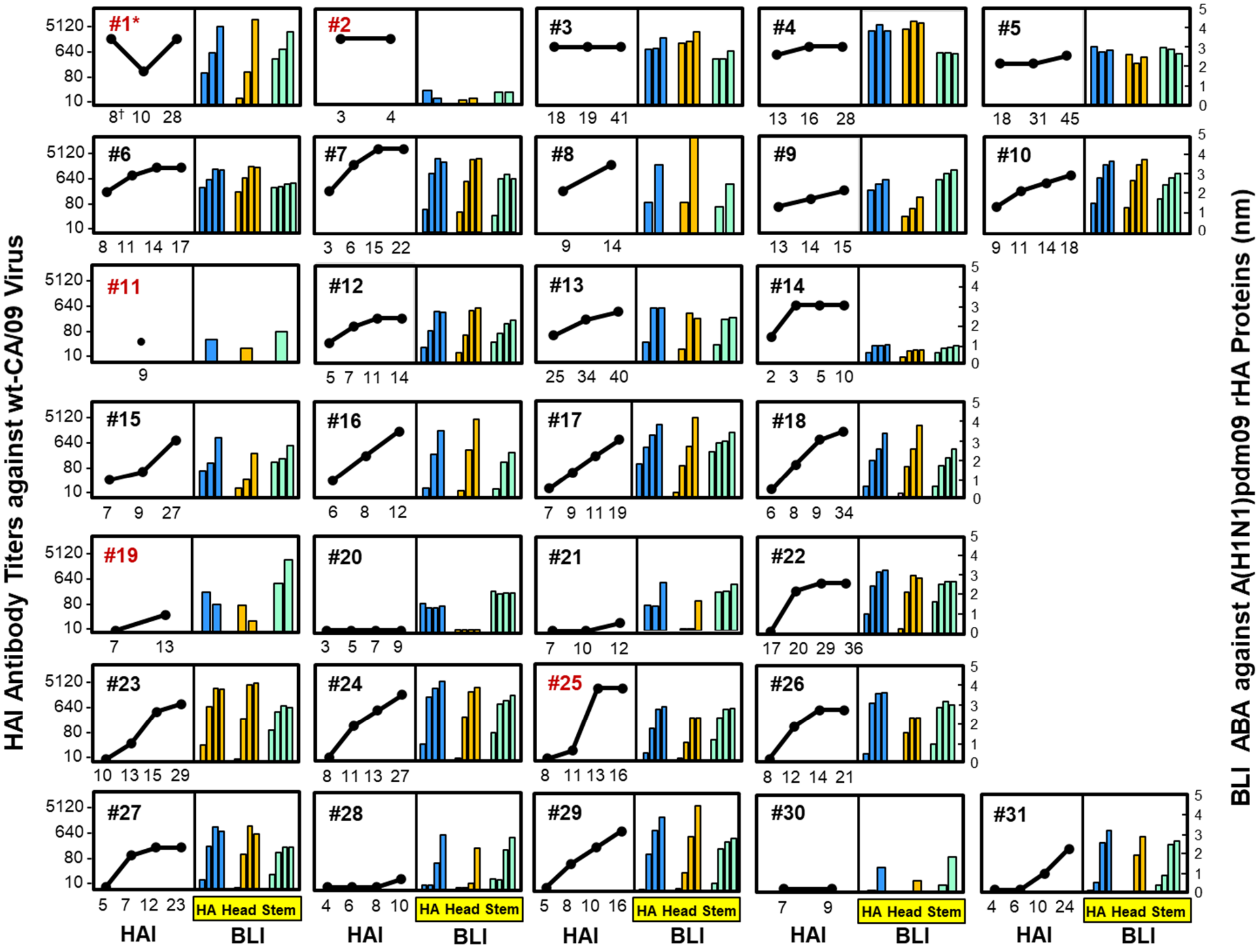

Supplementary Figure 1. HAI antibody responses and anti-rHA antibody binding activities against A(H1N1)pdm09 virus antigens. Patients (n=31) were infected with influenza A(H1N1)pdm09 virus between 2009 and 2011. Serum samples collected 2 to 45 dpo were tested by HAI assays using wt-CA/09 virus and BLI assays using rHA protein from CA/09, rHA-head from CA/09, and rHA-stem from A/Michigan/45/2015 A(H1N1)pdm09 IAV. HAI antibody titers are illustrated by black lines and BLI antibody binding activity (ABA) are illustrated by colored bars: blue for anti-rHA ABA, orange for anti-rHA-head ABA, cyan for anti-rHA-stem ABA. *Patient number (fatal patients in red). †Serum collection days post-symptom onset (dpo). We completed 3 independent HAI assays and 2 BLI assays.

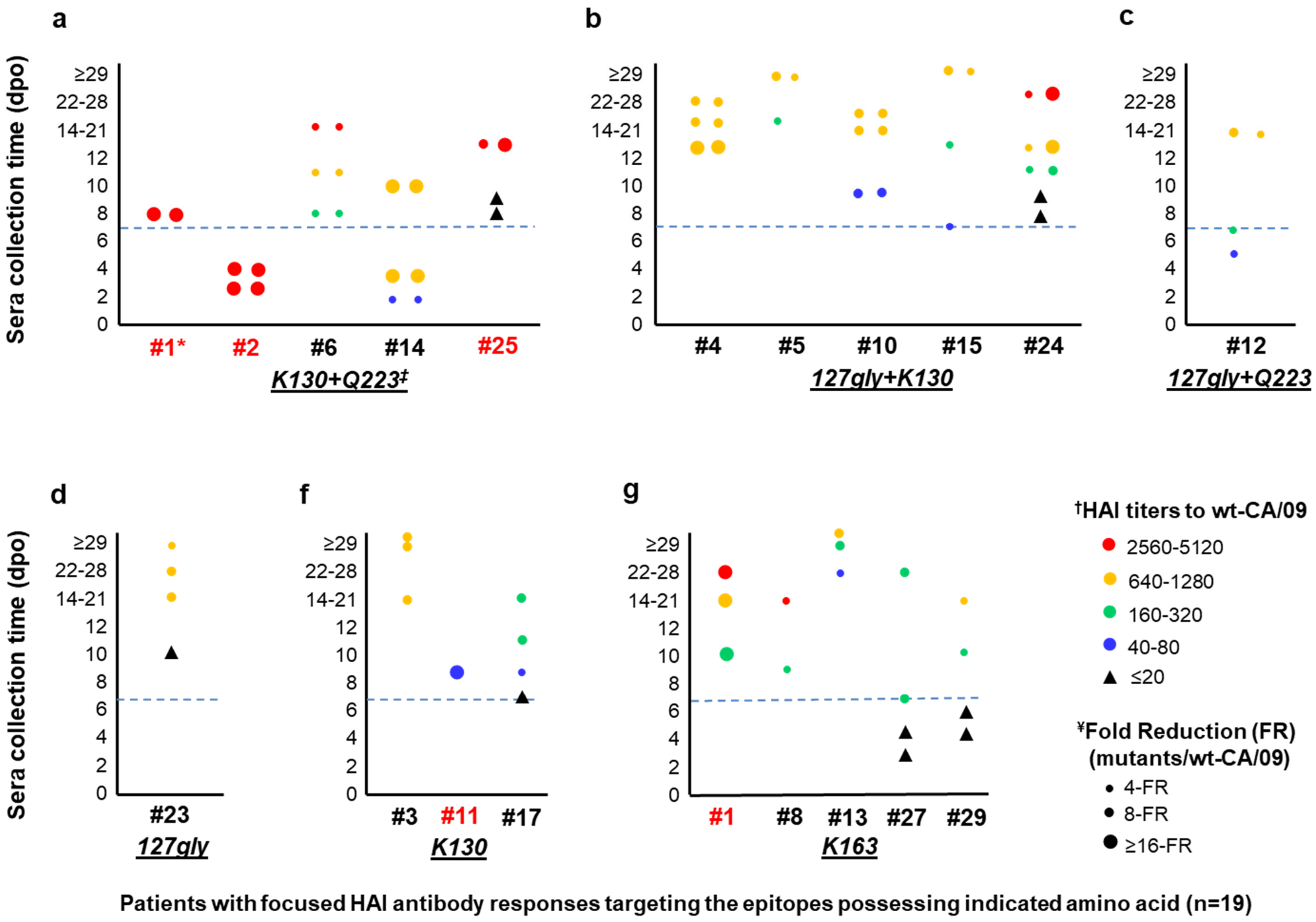

Supplementary Figure 2. Determination of immunodominance of HAI antibody responses and epitope mapping. Patients (n=19) were tested by HAI assays using 6 viruses: wt-CA/09, RG-K163Q, X-179A (Q223R), MX/09 (Q223QR), RG-130del, and RG-127gly (see Table 3 and Supplementary Table 1). †HAI antibody titers to wt-CA/09 were shown in black triangle (≤20) or colored dots: blue (40-80), green (160-320), orange (640-1280), and red (2560-5120) at the indicated days post symptom onset (dpo). ‡Key contact aa of the focused HAI antibody in each patient are determined as virus-mutants causing ≥4-fold reduction in HAI antibody titers compared to wt-CA/09. ¥levels of focused responses (fold reduction) are expressed by the sizes of dots. We completed 3 independent HAI assays.

Supplementary Table 1. Recombinant HA1 (rHA1) and RG viruses used for epitope mapping of focused antibody response

rHA1 used in BLI assays	Antigenic sites and/or RBS		Viruses used in HAI assays	
AA difference as compared to HA1-wt			Viruses	AA difference compared to CA/09 HA1
N125D	Sa			
D127T			RG-127gly	D127N, N129T
N129D				
K130E	RBS			
130del	RBS		RG-130del	130 deletion, S183ST (ST mixture)
K130N	RBS			
K142S	Ca2			
G155K	Sa			
N156D	Sa			
K163Q	Sa		RG-K163Q	K163Q
S183P				
T184K	Sb			
S190N	Sb			
L191K	RBS, Sb			
Q223R	RBS		MX/09<br>X-179A	Q223RQ (RQ mixture), P83S<br>Q223R, K209T

Supplementary Table 2. Determination of immunodominance of anti-HA-head binding antibody responses and epitope mapping

Patient		% ABA reduction against rHA1-mutants compared to rHA1-wt†															
no	dpo*	HA1-wt	N125D	D127T	N129D	K130E	130del	K130N	K142S	G155K	N156D	K163Q	S183P	T184K	S190N	L191K	Q223R
#1	8	NT1	NT1	NT1	NT1	NT1	NT1	NT1	NT1	NT1	NT1	NT1	NT1	NT1	NT1	NT1	NT1
	14	100	NT2	57	NT2	NT2	NT2	NT2	112	116	NT2	40	125	121	NT2	NT2	93
	21	100	NT2	63	NT2	NT2	NT2	NT2	104	105	NT2	37	99	103	NT2	NT2	89
#2	3	NT1	NT1	NT1	NT1	NT1	NT1	NT1	NT1	NT1	NT1	NT1	NT1	NT1	NT1	NT1	NT1
	4	NT1	NT1	NT1	NT1	NT1	NT1	NT1	NT1	NT1	NT1	NT1	NT1	NT1	NT1	NT1	NT1
#3	18	100	85	99	88	41	39	48	61	89	84	85	87	57	53	44	94
	41	100	76	96	90	62	58	68	85	88	88	93	82	78	74	63	91
#4	13	100	92	93	82	45	51	53	127	95	115	109	105	100	75	73	102
	28	100	93	92	88	47	53	52	120	95	112	105	108	97	73	67	103
#5	31	100	37	57	50	28	29	43	73	31	44	82	98	95	78	87	61
	45	100	40	57	56	25	31	45	86	38	44	100	105	103	82	86	85
#6	8	100	87	97	91	39	62	64	114	113	107	120	80	48	104	52	46
	14	100	91	93	92	49	64	65	92	95	92	102	65	43	83	40	53
#7	3	100	97	147	82	56	129	80	1	110	82	112	116	135	77	41	104
	15	100	91	96	88	71	95	86	7	90	84	94	96	98	75	60	88
	22	100	91	104	83	64	103	83	2	98	87	105	106	107	76	55	97
#8	9	NT2	NT2	NT2	NT2	NT2	NT2	NT2	NT2	NT2	NT2	NT2	NT2	NT2	NT2	NT2	NT2
	14	100	61	71	91	75	89	89	83	90	71	62	99	93	72	72	94
#9	13	NT2	NT2	NT2	NT2	NT2	NT2	NT2	NT2	NT2	NT2	NT2	NT2	NT2	NT2	NT2	NT2
	15	100	72	70	79	51	63	68	70	79	78	89	95	95	81	73	64
#10	9	100	69	69	83	42	45	78	95	120	95	113	129	100	98	101	109
	18	100	88	93	79	54	68	66	69	91	84	91	104	87	77	78	90
#11	9	NT2	NT2	NT2	NT2	NT2	NT2	NT2	NT2	NT2	NT2	NT2	NT2	NT2	NT2	NT2	NT2
#12	5	100	63	94	53	0	69	6	19	81	63	91	28	59	22	0	22
	7	100	84	111	72	0	58	15	47	106	87	101	43	82	56	2	57
	14	100	89	98	79	13	57	36	63	103	87	97	74	98	64	22	66
#13	25	100	28	63	88	76	94	93	114	105	84	38	117	106	95	98	63
	40	100	27	70	92	81	95	91	102	109	98	30	112	100	93	94	103
#14	2	NT1	NT1	NT1	NT1	NT1	NT1	NT1	NT1	NT1	NT1	NT1	NT1	NT1	NT1	NT1	NT1
	10	NT1	NT1	NT1	NT1	NT1	NT1	NT1	NT1	NT1	NT1	NT1	NT1	NT1	NT1	NT1	NT1
#15	27	100	83	81	62	42	58	63	88	109	88	120	110	106	83	87	102
#16	12	100	59	78	96	60	69	79	98	116	109	74	136	119	113	112	113
#17	11	100	88	104	68	18	33	26	81	75	111	91	117	70	15	13	33
#18	11	100	81	64	83	56	76	84	94	59	68	80	99	97	87	92	82
	34	100	71	68	82	62	78	87	96	84	83	83	108	102	93	97	91
#19	7	NT2	NT2	NT2	NT2	NT2	NT2	NT2	NT2	NT2	NT2	NT2	NT2	NT2	NT2	NT2	NT2
	13	NT1	NT1	NT1	NT1	NT1	NT1	NT1	NT1	NT1	NT1	NT1	NT1	NT1	NT1	NT1	NT1
#20	9	NT1	NT1	NT1	NT1	NT1	NT1	NT1	NT1	NT1	NT1	NT1	NT1	NT1	NT1	NT1	NT1
#21	12	NT2	NT2	NT2	NT2	NT2	NT2	NT2	NT2	NT2	NT2	NT2	NT2	NT2	NT2	NT2	NT2
#22	20	100	80	101	81	68	119	100	71	90	83	81	105	129	69	71	90
	29	100	82	109	75	58	126	102	63	101	86	97	115	139	70	73	83
#23	15	100	99	101	100	71	101	89	99	122	103	123	112	112	92	90	108
	29	100	91	90	91	65	93	85	77	92	82	97	98	93	73	72	83
#24	13	100	81	93	80	30	30	52	51	97	65	96	99	104	59	38	82
	27	100	91	94	89	33	36	68	59	114	92	115	117	118	80	49	102
#25	8	NT1	NT1	NT1	NT1	NT1	NT1	NT1	NT1	NT1	NT1	NT1	NT1	NT1	NT1	NT1	NT1
	13	100	92	112	86	64	76	72	82	98	96	103	104	103	92	86	91
#26	14	100	84	77	93	61	76	89	91	66	54	89	103	95	81	88	87
	21	100	77	72	88	59	71	88	90	68	60	90	103	95	81	94	78
#27	12	100	62	78	94	86	97	102	110	109	104	47	113	107	106	113	103
	16	100	65	72	94	83	93	97	95	89	89	43	104	97	90	83	84
#28	10	NT2	NT2	NT2	NT2	NT2	NT2	NT2	NT2	NT2	NT2	NT2	NT2	NT2	NT2	NT2	NT2
#29	16	100	55	82	87	55	68	70	83	103	55	66	84	78	82	77	100
#30	9	NT1	NT1	NT1	NT1	NT1	NT1	NT1	NT1	NT1	NT1	NT1	NT1	NT1	NT1	NT1	NT1
#31	10	100	90	89	89	78	81	84	100	110	99	83	116	98	97	92	70
	24	100	91	88	89	75	87	86	79	106	96	102	106	89	92	80	86

†Anti-HA-head (anti-HA1) antibody binding activity (ABA) were determined by BLI assays using a rHA1-wt (HA1 from wt-CA/09) and the 15 indicated rHA1-mutants.
*Serum collection days post-symptom onset (dpo); NT1, not tested due to low ABA (≤0.6 nm). NT2, not tested due to insufficient volume of serum.

More than 50% reduction of ABA to anti-rHA1-mutant(s) compared to rHA1-wt in blue; fatal cases in red.

Supplementary Table 3. HAI antibody specificity in A(H1N1)pdm09 virus-infected ICU patients

Patient	Sera†		wt-CA/09	USSR/77	ENG/80	TW/86	TX/91	NC/99	SI/06	BR/07	BR/10	BR/60
no. (#)	dpo‡	dpicu‡	A(H1N1)pdm09	A(H1N1)	A(H1N1)	A(H1N1)	A(H1N1)	A(H1N1)	A(H1N1)	A(H1N1)	A(H3N2)	B
#1*	8	2	2560	80	40	20	40	160	<	160	<	1280
	10	4	160	40	40	<	<	<	<	<	<	<
	28	22	2560	2560	2560	320	320	20	20	<	<	<
#2	3	3	2560	320	160	40	160	640	20	320	40	1280
	4	4	2560	160	80	40	160	640	20	320	40	1280
#3	18	1	1280	2560	1280	1280	320	20	20	20	<	20
	35	18	1280	1280	640	640	320	20	20	20	<	20
	41	24	1280	1280	640	640	640	20	<	<	<	40
#4	13	7	640	40	80	1280	2560	<	160	<	<	<
	28	22	1280	80	160	2560	2560	<	320	<	<	<
#5	18	2	320	<	<	<	<	<	<	<	<	<
	31	15	640	<	<	<	<	<	<	<	<	<
	45	29	640	<	20	<	20	<	<	20	<	<
#6	8	1	320	320	160	320	160	<	<	<	40	20
	11	4	1280	1280	1280	1280	640	20	<	20	160	40
	14	7	2560	1280	1280	1280	1280	20	20	20	320	40
#7	3	2	320	<	<	<	<	<	<	<	<	20
	8	7	12800	<	<	<	<	<	<	<	<	40
	22	21	12800	<	<	<	<	<	<	<	<	20
#8	9	2	160	80	40	<	<	<	<	<	<	<
	14	7	5120	2560	1280	160	160	40	80	40	<	<
#9	13	2	80	20	20	20	20	20	<	20	80	<
	15	4	320	80	40	40	80	40	20	80	80	<
#10	9	2	80	<	20	<	20	20	<	<	20	20
	18	11	1280	<	20	320	320	<	20	<	<	<
#11	9	2	80	<	<	80	80	20	40	<	20	<
#12	5	1	80	<	<	80	80	<	<	<	<	<
	7	3	160	<	<	160	160	<	<	<	<	<
	14	10	640	<	<	640	640	<	20	<	<	<
#13	25	5	80	20	20	<	<	<	<	<	<	<
	34	14	640	160	160	20	20	20	20	<	20	20
	40	20	640	320	160	40	20	40	20	<	20	20
#14	2	2	80	20	<	40	40	<	<	<	<	<
	4	4	1280	80	80	40	80	320	20	80	20	160
#15	13	8	320	<	<	40	40	<	<	<	<	<
	27	22	640	20	40	320	320	<	<	<	<	<
#16	6	1	40	<	<	20	20	<	<	<	<	<
	8	3	320	40	20	80	80	<	20	<	<	<
	12	7	640	160	80	640	640	80	160	80	20	<
#17	9	5	80	80	80	320	320	160	160	80	<	<
	11	7	320	320	160	1280	1280	320	640	320	<	<
	19	15	1280	1280	1280	2560	2560	1280	2560	1280	<	<
#18	8	4	160	40	40	80	80	80	80	<	160	20
	11	7	2560	640	320	320	320	160	160	40	160	20
	34	30	2560	640	320	320	320	160	320	80	160	20
#22	20	6	320	<	80	40	40	80	160	160	<	<
	36	22	640	<	80	80	40	320	640	640	<	<
#23	15	7	640	<	<	<	<	<	<	<	20	40
	29	21	1280	<	<	<	<	20	<	<	40	80
#24	8	1	<	<	<	<	<	<	<	<	<	<
	27	20	2560	20	20	640	1280	<	40	<	<	20
#25	8	2	<	<	<	<	<	<	<	<	40	<
	13	7	5120	160	160	80	160	640	40	320	80	640
#26	14	8	1280	40	40	20	20	20	<	<	<	<
	21	15	640	20	40	20	<	<	<	<	<	<
#27	5	4	<	<	<	<	<	<	<	<	<	40
	7	6	160	80	80	20	20	<	20	<	<	20
	23	22	320	160	80	20	20	<	20	<	<	20
#29	10	7	320	160	160	40	40	<	<	<	40	<
	16	13	1280	1280	640	160	160	80	40	40	320	<
#31	10	7	40	40	40	<	<	20	<	<	<	<
	24	21	320	160	160	20	20	20	<	<	<	<

† Sera from 26 patients, who had HAI antibody titers of ≥80, were tested by HAI assays with the 10 indicated viruses.
* Fatal patients in red; <, HAI titers below 20; ‡ dpo, days post-symptom onset; ‡ dpicu, days post-ICU admission.

Supplementary Table 4. IgM, IgA, and IgG subclass antibody responses to CA/09 HA

Patient			Sera†		ELISA titers					
no. (#)	Birth Year	Age (y)	dpo	dpicu	IgM	IgA	IgG1	IgG2	IgG3	IgG4
#1*	1962	47	8	2	200	800	**3,200**	<	<	<
			21	15	400	**40000‡**	**40,000**	400	1600	800
#2	1969	40	3	3	<	400	200	<	100	<
			4	4	<	400	200	<	100	<
#3	1966	43	18	1	<	**25,600**	6,400	200	400	<
			19	2	<	**25,600**	6,400	200	400	<
#4	1982	27	13	7	200	12,800	**80,000**	<	3,200	<
			28	22	100	12,800	**80,000**	200	3,200	<
#5	1956	53	31	15	200	**6,400**	**3,200**	200	**3,200**	400
#6	1963	46	8	1	<	**25,600**	1,600	200	400	200
			17	10	200	**80,000**	6,400	800	1,600	800
#7	1959	50	3	2	<	800	800	<	200	<
			15	14	200	**80,000**	**160,000**	<	12,800	400
#8	1963	46	9	2	<	800	**3,200**	200	**3,200**	<
			14	7	400	12,800	**80,000**	12,800	**160,000**	<
#9	1986	23	13	2	**1,600**	**800**	200	<	400	<
			15	4	**6,400**	**3,200**	1,600	<	**3,200**	<
#10	1956	53	9	2	<	**6,400**	1,600	<	200	<
			18	11	800	10,000	**40,000**	200	1,600	100
#11	1992	17	9	2	<	400	**1,600**	<	200	<
#12	1956	53	5	1	<	**1,600**	200	200	100	<
			14	10	<	**12,800**	1,600	200	400	<
#13	1980	29	25	5	<	**1,600**	**1,600**	<	<	<
			40	20	200	**12,800**	**12,800**	<	<	<
#14	1974	35	2	2	<	400	200	<	<	<
			10	10	<	400	200	<	<	<
#15	1952	57	7	2	100	800	400	<	<	<
			27	22	3,200	**12,800**	**12,800**	<	400	<
#16	1958	51	6	1	<	800	200	<	200	<
			12	7	200	**25,600**	6,400	200	6,400	400
#17	1985	24	7	3	<	200	800	<	<	<
			19	15	800	6,400	**40,000**	3,200	1,600	<
#18	1969	40	6	2	<	400	800	<	100	<
			11	7	<	**25,600**	**25,600**	800	3,200	<
#19	1967	42	7	3	<	**6,400**	**3,200**	<	400	100
			13	9	400	**6,400**	**3,200**	200	1,600	200
#20	1957	52	3	1	100	400	200	200	100	100
			9	7	200	800	400	200	100	100
#21	1953	56	7	4	<	<	800	<	<	<
			12	9	<	**3,200**	**6,400**	<	<	<
#22	1954	55	17	3	100	800	400	<	200	<
			29	15	400	**40,000**	**40,000**	1,600	800	<
#23	1978	31	10	2	<	400	100	<	800	100
			22	14	<	**12,800**	**12,800**	200	**12,800**	800
#24	1954	55	8	1	<	200	100	<	<	<
			27	20	<	800	**3,200**	<	800	<
#25	1944	65	8	2	<	200	200	<	<	<
			16	10	200	**6,400**	**12,800**	800	100	<
#26	1953	58	8	2	200	400	<	<	<	<
			14	8	6,400	**160,000**	20,000	200	12,800	<
#27	1968	41	5	4	<	200	400	<	100	<
			7	6	100	**1,600**	**3,200**	<	100	<
#28	1973	36	4	2	<	800	400	100	100	<
			10	8	100	**12,800**	**12,800**	200	200	<
#29	1970	39	5	2	200	400	200	<	100	<
			16	13	400	**12,800**	**12,800**	200	800	<
#30	1987	22	7	2	100	200	200	<	<	<
			9	4	100	400	**1,600**	<	<	<
#31	1957	52	5	2	<	400	<	100	100	100
			17	14	200	**40,000**	6,400	400	400	200

†Sera from 31 patients were collected 2-40 days post-symptom onset (dpo) and 1-22 days post ICU admission (dpicu). For each sample, we completed at least two independent ELISA assays using rHA from CA/09 virus.
‡Titers in bold display the highest titers (>800) that show ≥4-fold higher than others.
*Fatal patients in red; <, ELISA titers below 100.

References

1. Yang H, Carney P, Stevens J. 2010. Structure and Receptor binding properties of a pandemic H1N1 virus hemagglutinin. PLoS Curr 2:RRN1152.
2. Yassine HM, Boyington JC, McTamney PM, Wei CJ, Kanekiyo M, Kong WP, Gallagher JR, Wang L, Zhang Y, Joyce MG, Lingwood D, Moin SM, Andersen H, Okuno Y, Rao SS, Harris AK, Kwong PD, Mascola JR, Nabel GJ, Graham BS. 2015. Hemagglutinin-stem nanoparticles generate heterosubtypic influenza protection. Nat Med 21:1065-70.
